# A discussion of an issue relating to sample sizes of exact phase II trial designs

**DOI:** 10.1186/1745-6215-14-S1-P70

**Published:** 2013-11-29

**Authors:** Andrew Howman

**Affiliations:** 1 University of Birmingham, Birmingham, UK

## 

A'Hern's and Jung's single stage designs are phase II trial designs based on dichotomous outcomes. They are used in the single arm and randomised settings respectively. Decision rules at the end of the study are based on a required number of either treatment successes or extra successes on the treatment arm. They are exact designs in that the power and alpha can be calculated directly using binomial probabilities. Typically one would choose the power and alpha and then pick the smallest sample size that meets these restrictions. The discrete nature of the set of possible decision rules results in a somewhat erratic behaviour of the calculated sample sizes. In some situations, small changes to the design parameters can result in important reductions in sample size. Possible changes include relaxing one's alpha / beta slightly, or using intermediate stopping rules (eg Jung's / Simon's minimax designs). When designing these studies it is helpful to be aware of these issues and take account of them, so as to keep the sample sizes as small as possible.

